# Neighborhood socio-economic disadvantage and race/ethnicity as predictors of breast cancer stage at diagnosis

**DOI:** 10.1186/1471-2458-13-1061

**Published:** 2013-11-11

**Authors:** Yvonne N Flores, Pamela L Davidson, Terry T Nakazono, Daisy C Carreon, Cynthia M Mojica, Roshan Bastani

**Affiliations:** 1 UCLA Department of Health Policy and Management, Center for Cancer Prevention and Control Research, Fielding School of Public Health and Jonsson Comprehensive Cancer Center, 650 Charles Young Drive S., A2-125 CHS, Box 956900-6900, Los Angeles, CA 90095, USA; 2 Unidad de Investigación Epidemiológica y en Servicios de Salud, Instituto Mexicano del Seguro Social, Blvd. Benito Juárez No. 31 Col. Centro, Cuernavaca, Morelos C.P. 62000, México; 3 UCLA Clinical and Translational Science Institute, CTSI-Evaluation Sciences, 10880 Wilshire Blvd., Ste. 500, Los Angeles, CA 90024, USA; 4 UCLA School of Nursing, 10880 Wilshire Blvd., Ste. 500, Los Angeles, CA 90024, USA; 5 UCLA Department of Health Policy and Management, 650 Charles Young Dr. S., 31-269 CHS Box 951772, Los Angeles, CA 90095-1772, USA; 6 UCLA David Geffen School of Medicine, Division of General Internal Medicine & Health, Services Research, 911 Broxton Plaza, Ste. 101, Box 951736, Los Angeles, CA 90095-1736, USA; 7 Institute for Health Promotion Research, Department of Epidemiology and Biostatistics, School of Medicine, Cancer Therapy & Research Center, the University of Texas Health Science Center at San Antonio, 7411 John Smith Dr., Suite 1000, San Antonio, TX 78229, USA

**Keywords:** Breast cancer, Stage at diagnosis, Screening, Disparities

## Abstract

**Background:**

This study investigated the role of key individual- and community-level determinants to explore persisting racial/ethnic disparities in breast cancer stage at diagnosis in California during 1990 and 2000.

**Methods:**

We examined socio-demographic determinants and changes in breast cancer stage at diagnosis in California during 1990 and 2000. In situ, local, regional, and distant diagnoses were examined by individual (age, race/ethnicity, and marital status) and community (income and education by zip code) characteristics. Community variables were constructed using the California Cancer Registry 1990-2000 and the 1990 and 2000 U.S. Census.

**Results:**

From 1990 to 2000, there was an overall increase in the percent of in situ diagnoses and a significant decrease in regional and distant diagnoses. Among white and Asian/Pacific Islander women, a significant percent increase was observed for in situ diagnoses, and significant decreases in regional and distant diagnoses. Black women had a significant decrease in distant -stage diagnoses, and Hispanic women showed no significant changes in any diagnosis during this time period. The percent increase of in situ cases diagnosed between 1990 and 2000 was observed even among zip codes with low income and education levels. We also found a significant percent decrease in distant cases for the quartiles with the most poverty and least education.

**Conclusions:**

Hispanic women showed the least improvement in breast cancer stage at diagnosis from 1990 to 2000. Breast cancer screening and education programs that target under-served communities, such as the rapidly growing Hispanic population, are needed in California.

## Background

Breast cancer stage at diagnosis is an important determinant of outcomes, and is directly related to survival and mortality [1-4]. Stage at diagnosis can be used to report patterns of disease, document improvements in diagnosis and therapy [5], help identify and target interventions in high-risk subgroups [6], and assist policymakers in estimating and allocating resources [7,8]. Factors related to stage at diagnosis are similar to those associated with mammography utilization and breast cancer mortality. Women with low education and income levels, who belong to certain racial/ethnic groups, are uninsured or underinsured, and have limited access to medical care are less likely to be screened [9,10], more likely to have breast cancer detected at an advanced stage [11,12], and less likely to survive [10,13].

Much of breast cancer research has focused on individual-level determinants (age at diagnosis, race/ethnicity, socioeconomic class, and health insurance status) [14-16]. Less is reported about the effects of community-level determinants (health policy, health care delivery system, and community risk factors) and the extent to which they contribute to observed geographic variation in breast cancer stage at diagnosis. Prior work by the authors and other researchers has examined geographic variation in breast cancer stage at diagnosis and the influence of contextual variables, including community risk factors, physician supply, and HMO penetration [8,17-19]. The results of these studies indicate that community-level predictors are important. Women who reside in neighborhoods with more recent immigrants and a greater percentage of persons living below the federal poverty level and who are less educated, have a lower probability of using mammography services and being diagnosed at an earlier stage [8,20-22]. In California, lower percentages of early diagnosed breast carcinomas have also been found in non-urban areas characterized by greater distances, lower population density, and lower household incomes [23].

The degree to which socioeconomic status and urbanization contribute to the regional variation of breast cancer incidence in California has also been examined. Data from 1988-1997 show greater rates of breast cancer in urban versus non-urban areas, peaking among block groups with a high socioeconomic status [24]. Several studies have reported that breast cancer incidence is higher among women who are more educated and have a greater income [10,25-27]. To determine whether this effect was due to individual- or community-level factors, Robert et al. conducted a study controlling for education and other individual-level risk factors (age, mammography use, family history of breast cancer, reproductive factors, alcohol intake, and body mass index). Results indicated that women living in communities with the highest socioeconomic status had greater odds of having breast cancer than women who lived in the communities with the lowest socioeconomic status [25].

These studies conclude that community socioeconomic status and urbanity are not simply proxies for individual-level socioeconomic status and that living in certain communities may be associated with an increased risk of breast cancer. However, these studies looked at aggregate breast cancer cases and did not differentiate based on breast cancer stage at diagnosis. In response to persistent racial/ethnic and geographic disparities in mammography use and breast cancer mortality, this study sought to provide additional evidence about the critical influence of individual- and community-level determinants on observed disparities in breast cancer stage at diagnosis in California. With that aim, we compared changes in breast cancer stage at diagnosis during 1990 and 2000 and examined some of the individual- and community-level socio-demographic determinants that have influenced these changes over time. We combined census and cancer registry data to assess if changes in breast cancer stage at diagnosis varied by race/ethnicity and zip code levels of income and education. This analysis allowed us to identify which types of communities in California have the least favorable breast stage at diagnosis outcomes, and should be targeted for breast cancer screening and education programs.

### Conceptual framework

**Figure 1:**
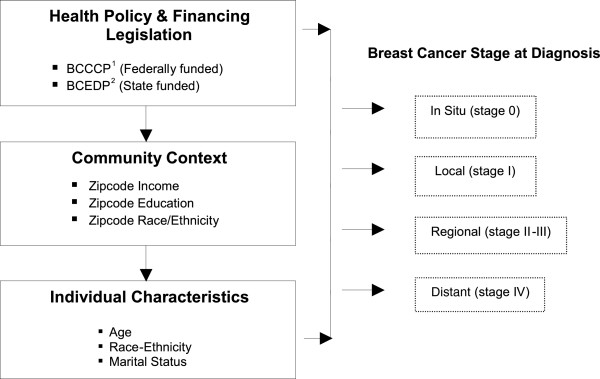
Factors associated with breast cancer stage at diagnosis*. * Adapted from Davidson et al. Cancer, 2005. ^1^ Breast and Cervical Cancer Control Program (BCCCP). ^2^ Breast Cancer Early Detection Program (BCEDP).

Figure 1 presents some of the factors that have been associated with breast cancer stage at diagnosis. This framework includes the specific individual- and community-level variables that were examined as part of this study. Community factors, such as the level of income and education in a particular zip code, can have an effect on a woman’s access to medical care and subsequent breast cancer stage at diagnosis. Individual characteristics such as age, race/ethnicity and marital status, are more proximal determinants of breast cancer stage at diagnosis. These specific variables were investigated because their data were available to our research team. These individual and community predictors were examined for each of the following progressive breast cancer screening diagnoses: in situ (stage 0), local (stage I), regional (stage II/III), and distant (stage IV).

Also included in the framework are the two breast cancer screening programs, which were sponsored by the Cancer Detection Section (CDS) of the California Department of Health Services, and were implemented in 1991: 1) the Breast Cancer Early Detection program (BCEDP) and 2) the Breast and Cervical Cancer Control Program (BCCCP). In 2002, both programs were combined and renamed Cancer Detection Programs: Every Women Counts (CDP:EWC). The CDP:EWC program provides free breast exams and mammograms to women who qualify, and offers breast and cervical cancer screening and diagnostic services to approximately 210,000 women each year [28]. Women seeking breast cancer screening and diagnostic services from these programs are required to meet the following criteria: 1) have a California address, 2) be aged 40 or older, 3) household income at, or below 200% of the Federal Poverty Level (FPL), and 4) be either uninsured or underinsured [28].

## Methods

### Study area and population and data

Data for this study were obtained from the California Cancer Registry and the 2000 United States (U.S.) Census. The California Cancer Registry is a statewide, population-based, cancer surveillance system that obtains information on all cancers diagnosed in California from medical facilities, which collect and report cancer data from their medical records and physicians who report information on cancer patients not referred to a medical facility. The California Cancer Registry provided data for all breast cancer cases diagnosed in 1990 and 2000. Data was extracted from the 2000 U.S. Census Summary File 3, which consists of 813 detailed tables of social, economic and housing characteristics compiled from a sample of approximately 19 million housing units (about 1 in 6 households) that received the Census 2000 long-form questionnaire.

### Individual variables

Information on the following individual characteristics was obtained from the California Cancer Registry: (1) age; (2) race/ethnicity (non-Hispanic white, non-Hispanic black, non-Hispanic Asian/Pacific Islander (PI), and Hispanic); (3) marital status (single, married/separated, widowed/divorced); and (4) breast cancer stage at diagnosis.

### Community variables

The community-level variables obtained from the 2000 U.S. Census Summary File 3 at the zip code level were: (1) income, defined as percentage of residents living below the 200% FPL; and (2) education, defined as percentage of residents who did not complete high school. These two variables were categorized into quartiles. The income categories were: (1) ≤ 16.0% (i.e. least poverty, or highest income quartile); (2) 16.1%-28.2%; (3) 28.3%-42.1%; and (4) ≥ 42.2% (i.e. most poverty or lowest income quartile). The education categories were: (1) ≤ 9.5% (i.e. lowest percentage of non-graduates, or highest education quartile); (2) 9.6%-17.4%; (3) 17.5%-29.8%; and (4) ≥ 29.9% (i.e. highest percentage of non-graduates, or lowest education quartile).

### Analyses

We restricted our analyses to women ages 40-64 with non-missing data for stage at diagnosis or Census-level variables. The California Cancer Registry originally contained n = 19,730 and n = 25,871 diagnosed breast cancer cases for the years 1990 and 2000, respectively. When this dataset was merged with the 2000 U.S. Census by zip code, we only used zip codes that met the following criteria: (1) breast cancer cases diagnosed as in-situ, local, regional or distant in both 1990 and 2000; and (2) non-missing values for federal poverty level and education (n = 1,011 zip codes). This resulted in n = 16,251 and n = 23,282 cases for 1990 and 2000, respectively. Our final sample size was n = 7,619 for 1990 and n = 11,967 for 2000, after eliminating women younger than 40 and older than 64.

First, we compared breast cancer stage at diagnosis, examining differences in 1990 and 2000 by individual characteristics: age, race/ethnicity and marital status, as well as two community-level characteristics: income (poverty level serving as a proxy) and education (Table 1). We then compared differences in breast cancer stage at diagnosis in 1990 and 2000 by community-level variables, for each racial/ethnic group (Table 2). For both tables, t-tests were used to test for significant differences in the percentage of breast cancer cases diagnosed at each stage between 1990 and 2000, across all individual and community-level variables. Besides testing each stage individually, we also conducted chi-square tests to assess changes in the distribution of all four stages simultaneously. Chi-square tests were also used to examine differences within each individual and community-level variable by year. Percentages based on counts with less than 16 individuals were not reported (shown as a ‘-‘ ) due to issues of unreliability. Tests of significance (t-tests and chi-square tests) were not shown if one or more comparison groups were based on these percentages. Finally, we used interaction terms derived from regression models to test for differences in the rates of change of diagnosed stages between 1990 and 2000 by individual and community characteristics.

**Table 1:** **Breast cancer stage at diagnosis for women 40-64 yrs., in 1990 and 2000 by individual and community characteristics**^**1**^

													**Percent difference between**				
	**1990**						**2000**						**1990 & 2000**^**2**^				**Overall**
	**N**	**In situ**	**Local**	**Regional**	**Distant**		**N**	**In situ**	**Local**	**Regional**	**Distant**		**In situ**	**Local**	**Regional**	**Distant**	**difference**
		**%**	**%**	**%**	**%**	**p-value**^**3**^		**%**	**%**	**%**	**%**	**p-value**^**4**^	**%**	**%**	**%**	**%**	**p-value**^**5**^
**Total**	7619	16.1	36.2	43.4	4.3		11967	19.7	36.2	40.9	3.2		3.6**	0.0	−2.5**	−1.1**	<0.0001
**INDIVIDUAL CHARACTERISTICS**																	
**Age**																	
40–49	2789	18.3	31.1	46.4	4.2	<0.0001	4080	20.8	31.3	45.2	2.7	<0.0001	2.5*	0.2	−1.2	−1.5**	0.0012
50–64	4830	14.8	39.1	41.7	4.4		7887	19.1	38.8	38.7	3.4		4.3**	−0.3	−3.0**	−1.0**	<0.0001
**Race/ethnicity**																	
White	5779	16.7	37.7	41.8	3.8	<0.0001	8323	19.9	38.6	38.6	2.9	<0.0001	3.2**	0.9	−3.2**	−0.9**	<0.0001
Black	460	13.3	26.3	52.4	8.0		767	17.5	28.3	49.3	4.9		4.0	2.4	−3.2	−3.2*	0.0342
Hispanic	840	15.3	31.8	48.0	4.9		1591	17.1	29.1	49.5	4.3		1.7	−2.7	1.5	−0.5	0.4016
Asian/Pacific Islanders	540	13.3	35.2	46.1	5.4		1286	22.8	34.5	40.4	2.3		9.5**	−0.7	−5.7*	−3.1**	<0.0001
**Marital status**^**6**^																	
Single	776	17.1	31.7	46.5	4.7	<0.0001	1798	17.6	35.6	42.3	4.5	<0.0001	0.5	3.9	−4.2*	−0.2	0.1955
Married/Separated	5159	16.6	37.6	42.3	3.5		7901	20.4	36.8	40.3	2.5		3.8**	−0.8	−2.0*	−1.0**	<0.0001
Widowed/Divorced	1549	12.9	34.7	46.0	6.4		1969	17.9	35.7	41.7	4.7		5.0**	1.0	−4.3*	−1.7*	<0.0001
**COMMUNITY CHARACTERISTICS**																	
**Zip code income level**^**7**^																	
≤ 16.0% (Less poverty)	2217	19.0	37.4	40.4	3.2	<0.0001	3655	21.3	39.7	36.8	2.2	<0.0001	2.3*	2.3	−3.6**	−1.0*	0.0013
16.1%–28.2%	2055	15.8	38.8	41.7	3.7		3299	19.4	37.5	40.4	2.7		3.6**	−1.3	−1.3	−1.0	0.0026
28.3%–42.1%	1744	15.3	36.6	43.8	4.3		2740	20.4	34.3	41.4	3.9		5.1**	−2.3	−2.4	−0.4	0.0003
≥ 42.2% (More poverty)	1603	13.4	30.7	49.4	6.5		2273	16.5	31.4	47.7	4.4		3.1**	0.7	−1.7	−2.1**	0.0023
**Zip code education level**^**8**^																	
≤ 9.5% (More educated)	2058	19.4	37.6	39.9	3.1	<0.0001	3291	21.6	39.2	37.0	2.2	<0.0001	2.2*	1.6	−3.0*	−0.8**	0.0163
9.6%–17.4%	2122	16.3	38.1	41.5	4.1		3483	19.9	37.6	39.7	2.8		3.6**	−0.5	−1.8	−1.3*	0.0008
17.5%–29.8%	1832	14.8	36.5	44.7	4.0		2859	19.3	35.5	41.6	3.6		4.5**	−1.0	−3.1*	−0.4	0.0011
≥ 29.9% (Less educated)	1607	13.1	31.5	49.0	6.4		2334	17.2	31.0	47.4	4.4		4.1**	−0.5	−1.7	−1.9**	0.0005

^1^These results were obtained using data from the California Cancer Registry and the U.S. Census. Only individuals residing in zip code areas that reported diagnoses in both 1990 and 2000 and had complete data for federal poverty level (FPL) and education in the 2000 U.S. Census (n = 1,011 zip code areas) were reported.

^2^Based on t-tests across breast cancer stage at diagnosis categories.

^3^Chi-square test of the difference between stages by variable category in 1990.

^4^Chi-square test of the difference between stages by variable category in 2000.

^5^Chi-square test of the overall difference between stages by variable category from 1990 to 2000.

^6^Total n-size for marital status in 1990 and 2000 is n = 7,484 and n = 11,668, respectively.

^7^Income quartiles, which represent the percentage of residents living below the 200% FPL, were derived from the 2000 U.S. Census sample estimates.

^8^Education quartiles, which represent the percentage of residents with less than a high school education, were derived from the 2000 U.S. Census sample estimates.

*p-value < 0.05.

**p-value < 0.01.

**Table 2:** **Breast cancer stage at diagnosis for women 40-64 yrs., in 1990 and 2000 by zip code income and education level**^1^**and race/ethnicity**^2^

													**Percent difference between**				
		**1990**						**2000**					**1990 & 2000**^**3**^				**Overall**
	**N**	**In situ**	**Local**	**Regional**	**Distant**		**N**	**In situ**	**Local**	**Regional**	**Distant**		**In situ**	**Local**	**Regional**	**Distant**	**difference**
		**%**	**%**	**%**	**%**	**p-value**^**4**^		**%**	**%**	**%**	**%**	**p-value**^**5**^	**%**	**%**	**%**	**%**	**p-value**^**6**^
**WHITE**	5779	16.7	37.7	41.8	3.8		8323	19.9	38.6	38.6	2.9		3.2**	0.9	−3.2**	−0.9**	<0.0001
**Zip code income level**^**7**^																	
≤ 16.0% (Less poverty)	1923	18.8	37.4	40.8	3.0	<0.0001	2924	21.1	40.9	35.8	2.2	<0.0001	2.3	3.5*	−5.0**	−0.8	0.0007
16.1%–28.2%	1660	16.3	39.8	40.3	3.6		2486	19.2	38.3	39.8	2.7		2.9*	−1.5	−0.5	−0.9	0.0553
28.3%–42.1%	1318	15.2	38.8	42.1	3.9		1898	21.1	36.6	39.0	3.3		5.9**	−2.2	−3.1	−0.6	0.0005
≥ 42.2% (More poverty)	878	15.0	32.9	46.3	5.8		1015	16.3	36.8	42.5	4.4		1.3	3.9	−3.8	−1.4	0.1217
**Zip code education level**^**8**^																	
≤ 9.5% (More educated)	1827	18.9	37.7	40.3	3.1	0.0006	2746	21.1	40.1	36.4	2.4	0.0046	2.1	2.5	−3.9**	−0.7	0.0156
9.6%-17.4%	1766	16.7	39.1	40.5	3.7		2748	19.9	38.5	39.0	2.6		3.1**	−0.6	−1.4	−1.1*	0.0138
17.5%–29.8%	1342	15.2	37.6	43.5	3.7		1858	18.8	37.8	40.2	3.2		3.6 **	0.2	−3.4	−0.4	0.0369
≥ 29.9% (Less educated)	844	14.2	35.1	44.9	5.8		971	18.8	36.5	40.3	4.4		4.6**	1.4	−4.6*	−1.4	0.0184

**BLACK**	460	13.3	26.3	52.4	8.0		767	17.5	28.3	49.3	4.9		4.0	2.4	−3.2	−3.2*	0.0342
**Zip code income level**^**7**^																	
≤ 16.0% (Less poverty)	33	─	─	─	─	─	80	─	35.0	46.2	─	─	─	─	─	─	─
16.1%–28.2%	81	─	29.6	50.6	─		152	17.1	36.8	40.8	─		─	7.2	−9.8	─	─
28.3%–42.1%	92	─	25.0	53.3	─		156	21.8	25.6	46.8	─		─	0.6	−6.5	─	─
≥ 42.2% (More poverty)	254	11.4	25.2	55.1	8.3		379	15.8	24.5	54.4	5.3		4.4	−0.7	−0.7	−3.0	0.2357
**Zip code education level**^**8**^																	
≤ 9.5% (More educated)	26	─	─	─	─	─	74	─	37.8	44.6	─	─	─	─	─	─	─
9.6%–17.4%	78	─	26.9	55.1	─		119	14.3	31.9	47.1	─		─	5.0	8.1	─	─
17.5%–29.8%	123	13.0	26.0	52.9	─		234	24.3	28.2	43.2	─		11.4**	2.2	−9.7	─	─
≥ 29.9% (Less educated)	233	10.7	26.2	54.1	9.0		340	14.1	25.0	55.3	5.6		3.4	−1.2	1.2	−3.4	0.2980

**ASIAN/PACIFIC**																	
**ISLANDERS**	540	13.3	35.2	46.1	5.4		1286	22.8	34.5	40.4	2.3		9.5**	−0.7	−5.7*	−3.1**	<0.0001
**Zip code income level**^**7**^																	
≤ 16.0% (Less poverty)	150	18.0	40.7	38.0	─	─	418	26.3	35.2	37.1	─	─	8.3*	−5.5	−0.9	─	─
16.1%–28.2%	163	13.5	32.5	49.7	─		366	22.1	35.5	40.2	─		8.6*	3.0	−9.5*	─	─
28.3%–42.1%	110	─	34.6	49.1	─		273	21.6	28.6	46.5	─		─	−6.0	−2.6	─	─
≥ 42.2% (More poverty)	117	─	32.5	48.7	─		229	18.8	38.4	39.7	─		─	5.9	−9.0	─	─
**Zip code education level**^**8**^																	
≤ 9.5% (More educated)	117	19.7	40.2	38.4	─	─	307	28.4	32.9	37.1	─	─	8.7	−7.3	−1.3	─	─
9.6%–17.4%	152	15.1	30.9	47.4	─		377	23.9	37.7	37.4	─		8.8*	6.7	−10.0*	─	─
17.5%–29.8%	142	─	39.4	46.5	─		335	20.3	31.3	44.5	─		─	−8.1	−2.0	─	─
≥ 29.9% (Less educated)	129	─	31.0	51.2	─		267	18.0	35.6	43.4	─		─	4.6	−7.8	─	─
**HISPANIC**	840	15.3	31.8	48.0	4.9		1591	17.1	29.1	49.5	4.3		1.7	−2.7	1.5	−0.5	0.4016
**Zip code income level**^**7**^																	
≤ 16.0% (Less poverty)	111	20.7	36.1	37.8	─	─	233	17.6	34.3	44.7	─	─	−3.1	−1.7	6.8	─	─
16.1%–28.2%	151	13.9	39.1	44.4	─		295	20.0	32.2	45.4	─		6.1	−6.9	1.0	─	─
28.3%–42.1%	224	18.7	29.9	46.9	─		413	16.2	30.8	46.7	6.3		−2.5	0.8	−0.1	─	─
≥ 42.2% (More poverty)	354	12.2	28.5	53.4	5.9		650	16.6	24.3	54.8	4.3		4.4*	−4.2	1.4	−1.6	0.1142
**Zip code education level**^**8**^																	
≤ 9.5% (More educated)	88	22.7	36.4	37.5	─	─	164	20.7	36.0	42.7	─	─	−2.0	−0.4	5.2	─	─
9.6%–17.4%	126	14.3	39.7	39.7	─		239	16.3	30.5	46.9	─		2.0	−9.2	7.2	─	─
17.5%–29.8%	225	16.4	33.8	45.8	─		432	17.6	32.6	45.2	4.6		1.1	−1.1	−0.6	─	─
≥ 29.9% (Less educated)	401	13.5	27.2	54.1	5.2		756	16.3	25.1	54.2	4.4		2.8	−2.1	0.1	−0.8	0.5320

^1^Quartiles derived from the 2000 U.S. Census sample estimates.

^2^These results were obtained using data from the California Cancer Registry and the U.S. Census. Only individuals residing in zip code areas that reported diagnoses in both 1990 and 2000 and had complete data for federal poverty level (FPL) and education in the 2000 U.S. Census were reported (n = 1,011 zip code areas).

^3^Based on t-tests across breast cancer stage at diagnosis categories.

^4^Chi-square test of the difference between stages by variable category in 1990.

^5^Chi-square test of the difference between stages by variable category in 2000.

^6^Chi-square test of the overall difference between stages by variable category from 1990 to 2000.

^7^Income quartiles, which represent the percentage of residents living below the 200% FPL, were derived from the 2000 U.S. Census sample estimates.

^8^Education quartiles, which represent the percentage of residents with less than a high school education, were derived from the 2000 U.S. Census sample estimates.

*p-value < 0.05.

**p-value < 0.01.

─Percentages not shown, since they were based on counts having less than 16 individuals; tests of significance for the categories with unreported percentages are also not reported.

## Results

Table 1 reports the percentage of women ages 40-64 who were diagnosed in each of the four breast cancer stages by individual- and community-level characteristics. From 1990 to 2000 there was a significant increase in the overall percentage of cases diagnosed as in situ and a significant decrease in the percentage of cases diagnosed as regional and distant. Not surprisingly, the overall percent distribution of the staged diagnoses between the two years was significantly different as well.

### Individual characteristics

Among women ages 40-49, there was a significant increase in the percent of in situ cases and a decrease in distant cases from 1990 to 2000. Among women ages 50-64, there was also a significant increase in the percent of in situ cases and a decrease in regional and distant cases. With respect to race/ethnicity, white and Asian/PI women had a significant increase in the percentage of in situ cases and a significant decrease in the percent of regional and distant cases. Among black women, there was a significant percent decrease in cases diagnosed at the distant stage, while Hispanic women showed no significant changes in any stage of diagnosis between 1990 and 2000. Regarding marital status, single women showed a significant decrease in the percentage of regional cases, while both married/separated and divorced/widowed groups showed a significant percent increase for in situ cases and a decrease in regional and distant cases. With the exception of Hispanics and single women, all age, race/ethnicity and marital status categories showed significant differences in the overall distribution of stages between the two years. Age, race/ethnicity and marital status also showed significant differences between categories by year as well. Compared to whites, Asian/PI women showed a significantly greater percent increase of diagnosed in situ cases and greater percent decrease of distant cases between 1990 and 2000, while Hispanics had a greater increase in percent cases diagnosed as regional, compared to whites (Data not shown).

### Community-level characteristics

Across all income and education categories, the percentage of in situ cases increased significantly from 1990 to 2000. The highest income and education quartiles showed a significant decrease in the percentage of regional and distant cases, while the lowest income and education categories had a significant decrease in the percent of distant cases. The second lowest and second highest education quartiles showed significant decreases in the percent of regional and distant cases, respectively. These differences between individual stages translated to significant differences in the overall distribution of the four stages between 1990 and 2000 across all income and education categories. As with the individual-level characteristics, significant differences between the community-level income and education categories were observed for each year as well (Table 1).

Table 2 presents breast cancer stage at diagnosis, by income and education for each race/ethnicity group. These analyses further explore the combined effects of individual-level race/ethnicity and the community-level determinants of income and education. For the non-white groups, several percent stage data were not reported because of insufficient sample sizes (n<16) as explained in the Methods section.

### White women

From 1990 to 2000, the two middle income quartiles showed a significant increase in the percentage of in situ cases, while the highest income quartile showed an increase in the percentage of local cases and a decrease in regional cases. Except for the highest education quartile, all other education groups showed a significant percent increase of in situ cases. Both the highest and lowest education quartiles showed significant decreases in the percentage of regional cases, while the second-highest education quartile showed a decrease in the percentage of distant cases. With the notable exception of the lowest income group, all income and education groups (with the second-highest income quartile being borderline at p = 0.055) showed significant differences in the distribution of diagnosed stages between the two years. Both community-level characteristics showed significant differences by year as well (Table 2).

### Black women

No significant differences were observed in percent stage at diagnosis by income level between 1990 and 2000. The second-lowest education quartile showed a significant increase in the percentage of cases diagnosed as in situ. Lowest income and lowest education – the only two quartiles with sufficient sample sizes across all stages – showed no significant changes in the distribution of diagnosed stages between 1990 and 2000 (Table 2).

### Asian/PI women

The two highest income quartiles showed a significant increase in the percentage of cases diagnosed as in situ from 1990 to 2000, while the second highest income quartile showed a significant percent decrease in diagnosed regional cases that were diagnosed between 1990 and 2000. For education, the second highest quartile showed a significant increase in the percentage of in situ cases and a significant decrease in the percentage of regional cases (Table 2).

### Hispanic women

Other than a significant increase in the percentage of cases diagnosed as in situ, in the lowest income quartile, no other significant changes occurred between 1990 and 2000 among the other income and education groups (Table 2).

## Discussion

This study explored specific socio-demographic determinants and changes in breast cancer stage at diagnosis in California from 1990 to 2000. Our results provide additional evidence that both community-level (e.g., zip code-level income and education) and individual characteristics (race/ethnicity and age) can be used to examine some of the disparities that were observed in early vs. advanced breast cancer stage at diagnosis during this time [8,29]. Other studies have looked at the effects of community-level determinants on the geographic variation of breast cancer cases detected in California [8,22,23,30,31]. This study further explores certain community-level variables, such as income and education, in addition to racial/ethnic differences by stage at diagnosis in California.

Our results indicate that the income and education levels of a community can be useful determinants of breast cancer stage at diagnosis disparities. Regardless of race/ethnicity, women in the quartile with the most poverty experience a lower percentage of breast cancer cases being detected at early stages and a higher percentage of cases detected at advanced stages, compared to women in the quartile with the least poverty. This finding supports the well-documented association between poverty and poorer breast cancer outcomes [18]. The same relationship is observed for education. Women who reside in communities where 90 percent of residents completed high school experience a higher percentage of early stage breast cancer diagnoses and a lower percentage of advanced-stage breast cancer, compared to women in areas where less than 70 percent completed high school.

Although income and education are highly correlated (0.8173 in 1990 and 0.8181 in 2000), we wanted to examine them as separate community-level variables. Among white women, we found a significant difference in the overall percent change from 1990 to 2000 in all breast cancer stages at diagnosis in the least educated quartile, but not in the quartile with the most poverty. We were unable to determine any other significant differences between income and education in the other race/ethnicity groups, due to inadequate sample sizes.

Harris et al. examined breast cancer stage at diagnosis among medically underserved women who received a mammogram through the Cancer Detection Section (CDS) of the California Department of Health Services-sponsored screening program [11]. Their finding that CDS program participants were more likely to be diagnosed at an advanced stage than non-participants supports our result that women in low-income communities have a lower percentage of early-stage breast cancer diagnoses and a higher percent of advanced-stage breast cancer diagnoses than women in higher income areas.

Substantial variation exists in California’s breast cancer rates, which are among some of the highest rates in the nation and world [24,32,33]. Although screening and survival rates have improved in California, geographic and racial/ethnic disparities continue to persist. From 1998 to 2008, breast cancer mortality declined 32 percent among non-Hispanic white women, 21 percent among Hispanic women, 15 percent among black women, but did not decline significantly for Asian/PI women [34]. Rates of screening also vary by race/ethnicity, and white women are more likely to have been screened recently than black, Hispanic, or Asian/PI women [34]. From 1994-2002, early stage breast cancer detections reached a plateau for all racial/ethnic groups in California, but remained far lower among black and Hispanic women than among non-Hispanic white and Asian/PI women [29].

Our findings indicate that from 1990 to 2000 a significant percentage increase was observed for in situ diagnoses among white and Asian/PI women, as well as significant decreases in the percentage of regional and distant cases diagnosed among white, black, and Asian/PI women. However, there was no significant increase in the percentage of early-stage diagnoses and no reduction in the percentage of advanced-stage diagnoses for Hispanic women in California during this time period. Several reasons might help to explain why Hispanic women showed the least improvement in breast cancer stage at diagnosis during this time, including barriers to screening access such as English-speaking ability [35], birthplace and low acculturation [36-38], as well as reduced health care and informational access [39,40]. A study by Crawley et al., found that perceived medical discrimination is also associated with lower breast cancer screening rates among racial and ethnic minorities in California [41]. Additionally, Hispanic women report that the most consistent predictors of adherence to mammography screening were health system factors, including having health insurance and a usual source of care [42]. According to the U.S. Census, 14 million of California’s 38 million residents are Hispanic, and 10.8 million are of Mexican origin (77%) [43]. California’s Hispanic population is projected to continue to grow rapidly, making it critical to identify ways to increase screening rates and prevent advanced stage breast cancer in this population.

This study has some important limitations. A shortcoming of our analyses is that we were restricted to the specific variables and data from 1990 to 2000 that was available to our research team. Because of this, we were unable to examine other individual and community-level determinants, or include data from other time periods. Also, although our final sample size of n = 7,619 for 1990 and n = 11,967 for 2000 was sufficiently large for the aggregate analyses, it was insufficient when the data was stratified by race/ethnicity and income or education. Additionally, we did not examine changes in the percentage of women who were missing stage at diagnosis. It is likely that missing stage would vary by individual and community-level characteristics and might not be equally distributed among the four stages. Another study limitation concerns the comparability of the 1990 U.S. Census statistics, which were reported by zip code, to the 2000 U.S. Census results, which were reported by Zip Code Tabulation Areas. Even though the codes may appear the same, the addresses and areas covered by these areas may not be the same. Despite these limitations, these are some of the few data sources available for these types of analyses.

## Conclusions

In summary, our findings support those of other studies that have identified high-risk populations, by examining the specific individual and community-level risk factors that are associated with the detection of either earlier or more advanced stages of breast cancer. Our results suggest that early detection breast cancer screening and education programs are critically needed in underserved communities, such as Hispanic women, who have higher rates of advanced-stage breast cancer diagnoses. The most recent California Cancer Registry report indicates that from 2005 to 2009 Asians/PI and white women had a greater percentage of in situ and local cases detected and a lower percentage of regional and distant cases detected than black and Hispanic women [44]. These more recent findings indicate that the racial/ethnic disparities in breast cancer stage at diagnosis we identified in our study continue to persist in California.

A greater effort should be made to implement breast cancer screening and education programs in California zip code areas that have a higher percentage of black and Hispanic women, as well as low-income areas and neighborhoods with a greater percentage of less-educated individuals. These groups had the lowest rates of early-stage breast cancer detection, and showed the least improvement from 1990 to 2000. Specific strategies, such as patient navigation, which are culturally tailored, system-based interventions that focus on individual barriers, could be used to target these populations [45]. Patient navigation has proven to improve mammography rates for inner-city, low-income, minority populations [46]. Other strategies, such as home-based group educational interventions delivered by *promotoras*, or bilingual community health educators, also appear to improve breast cancer screening practices among Hispanic women [47]. Interventions to reduce disparities in breast cancer stage at diagnosis among Hispanic women should also focus on increasing access to health services, including affordable insurance or free screening services and improving access to health care providers [48].

## Competing interests

The authors declare that they have no competing interests.

## Authors’ contributions

YF participated in the data analysis and drafted the manuscript. PD conceived the study, participated in its design and coordination and helped to draft the manuscript. TN participated in the design of the study and performed the statistical analysis. DC, TN, CM and RB helped analyze the data and significantly contributed to the manuscript preparation. All authors read and approved the final manuscript.

## Pre-publication history

The pre-publication history for this paper can be accessed here:

http://www.biomedcentral.com/1471-2458/13/1061/prepub
